# *Fragaria* Genus: Chemical Composition and Biological Activities

**DOI:** 10.3390/molecules25030498

**Published:** 2020-01-23

**Authors:** Radu Claudiu Fierascu, Georgeta Temocico, Irina Fierascu, Alina Ortan, Narcisa Elena Babeanu

**Affiliations:** 1 University of Agronomic Sciences and Veterinary Medicine of Bucharest, 59 Mărăști Blvd., 011464 Bucharest, Romania; radu_claudiu_fierascu@yahoo.com (R.C.F.); alina_ortan@hotmail.com (A.O.); narcisa.babeanu@gmail.com (N.E.B.); 2 Emerging Nanotechnologies Group, National Institute for Research & Development in Chemistry and Petrochemistry—ICECHIM Bucharest, 202 Spl. Independentei, 060021 Bucharest, Romania

**Keywords:** *Fragaria* genus, chemical composition, biological properties

## Abstract

The strawberries represent in our days one of the main fresh fruits consumed globally, inevitably leading to large amounts of by-products and wastes. Usually appreciated because of their specific flavor, the strawberries also possess biological properties, including antioxidant, antimicrobial, or anti-inflammatory effects. In spite of the wide spread of the *Fragaria* genus, few species represent the subject of the last decade scientific research. The main components identified in the *Fragaria* species are presented, as well as several biological properties, as emerging from the scientific papers published in the last decade.

## 1. Introduction

The production of different fruits all around the world exceeds millions of tons, depending on geographical zones, consumption, and growing traditions, inevitably leading to large amounts of by-products and wastes. *Fragaria* genus (*Rosaceae*), commonly known as strawberry, represents one of the most important food plants all over the world, with a double global production compared with all other fruit berries combined [1]. Their widespread use, primarily because of their flavor, can also lead to considerable benefits to human health. Among other characteristics, nonvisual properties like taste, nutritional values, or aroma make these fruits to be in the top of consumer preferences [2].

Known and consumed for thousands of years, *Fragaria* species are encountered throughout the northern hemisphere, as well as in some areas of South America [1]. Several authors present the historical consumption of strawberries in pre-Columbian sites, Picunche and Mapuche people (Chile), Romans or ancient China. [1,3,4,5] The exact number of accepted species of the genus remains a subject of debate, ranging from 22 [6] to 16 [7]. In addition, there are many hybrids and cultivars representing ploidy levels ranging from diploid (2*n* = 2x = 14) to decaploid (2*n* = 10x = 70), influencing the size of the fruits. Most of the research regarding the genus can be traced to the extraordinary work of Antoine Nicolas Duchesne, that offered botanical description, details on the history, cultivation, sex, and polyploidy of different species [5]. Generally speaking, all *Fragaria* species share some common characteristics: are low-growing perennials, with usually evergreen and trifoliolate leaves, insect-pollinated, with white actinomorphic flowers (usually 5-petalled). The main difference between species is represented by the animal-dispersed accessory aggregate fruits, in terms of color, shape, and achene (the 1-seeded simple fruits) and calyx positions at maturity. From the different composition and other characteristics of those fruits, also arise the potential commercial value of the species. Many cultivars are perennials that vary in their photoperiod needs, leading to varying harvesting times (June-bearers, ever-bearers, day-neutral) [8].

Among the 247 varieties known and listed, only few present commercial interest: *Fragaria x ananassa* Duchesne (octoploid hybrid-containing 56 chromosomes, known as garden strawberry, native to northern America, cultivated all over the world), and, to a lesser extent, *Fragaria vesca* L. (diploid species, known as wild strawberry, native to Northern hemisphere) and *Fragaria chiloensis* (L.) Mill. (octoploid species, known as Chilean strawberry, native to northern, pacific and southern America) [1].

As previously mentioned, the strawberries represent one of the most important fruit plants. Their production reached 9.22 million tones (world level) in 2017, the major producers being China (40.3% of total world production), United States (15.7%), Mexico (7.14%), Egypt (4.42%), Turkey (4.34%), Spain (3.9%), Republic of Korea (2.28%), Poland, Russian Federation, Morocco, Japan, Germany, United Kingdom, and Italy (between 1 and 2%) [9]. This mass-production invariably leads to large amounts of wastes, that can be further exploited in multiple areas, including medicine, cosmetics, and food industry [10]. The wastes are generated throughout the growth cycle (maturation, multiplication, and expansion), while the large-scale methods of cultivation (e.g., in fields, plastic tunnels., etc.) leads to large amounts of wastes from leaves, stolons, fruits, etc.

The present review paper aims to present the identified components in different species of the *Fragaria* genus, as well as their potential biological activities, as emerging from the scientific papers published in the past decade. The selection of the articles to be included in the present review was performed using the well-known data-bases (Scopus, Web of Science, ScienceDirect, and PubMed), using specific keywords (“composition”, “therapeutic”, “cytotoxic”, “anti*”—returning results for “antimicrobial”, “antifungal”, “anti-inflammatory”, etc.). The validation of the articles was performed manually (by reading the entire article) and in the present review were inserted only articles with significant contribution to the field of research.

## 2. Composition of *Fragaria* L. Genus

**Figure 1:**
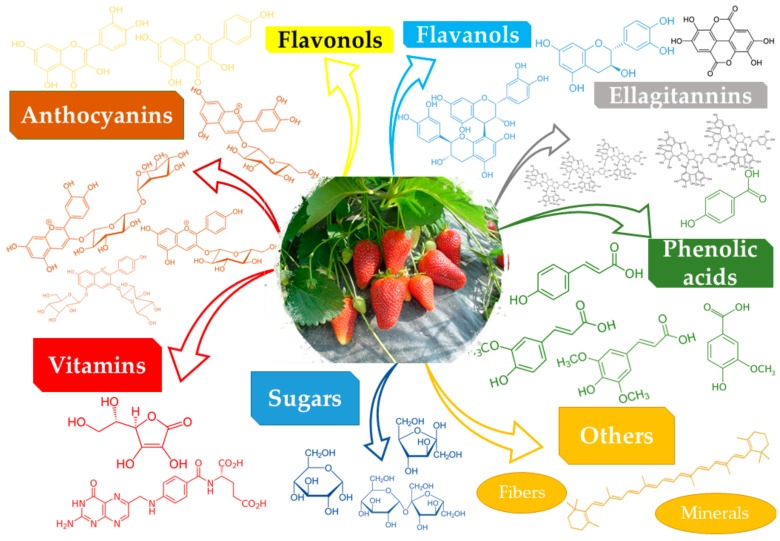
Main components *Fragaria* species identified according literature data.

Giampieri et al. [11] reviewed the composition of the strawberry (*Fragaria x ananassa*), while Morales-Quintana and Ramos [12] reviewed the composition and potential applications of the Chilean strawberry (*Fragaria chiloensis* (L.) Mill.), while the functional properties of the berries, in general, and of the strawberries, in particular, were reviewed by Jimenez-Garcia et al. [13]. As resulting from various literature studies [11,12,13,14,15], the general composition of the strawberries (in terms of major components) can be summarized in Table 1 (with a general image provided in Figure 1).

The presented composition varies with a series of factors, including the value of the cultivar, seasonal variation, and the degree of fruit ripeness. In the reviewed time period, several studies presented the evaluation of species belonging to *Fragaria* genus. Their main findings are presented in Table 2, while relevant studies are presented in the following paragraphs.

As the major bioactive constituents of *Fragaria* fruits are represented by anthocyanins, most of the literature studies are focused on their identification/quantification. Cerezo et al. [17] identified multiple anthocyanins and other phenolic compounds present in *Fragaria x ananassa* (*Camarosa* variety) puree, among which three of them (delphinidin-3-glucoside, peonidin-3-glucoside, cyanidin-3-galactoside) were proposed for the first time in the literature. A correlation between the cultivation system (classic/organic) and the composition of the strawberries was established by Crecente-Campo et al. [18]. The authors observed higher values of the identified anthocyanins and ascorbic acid (accompanied by a darker, redder color and a superior nutritional value) in the case of organic cultivated strawberries. The differences in terms of volatile esters composition between wild and cultivated strawberries were presented by Dong et al. [19]. The authors suggested that the composition in volatile esters (dominated in the case of *F. vesca* by acetate esters, and by ethyl hexanoate, in the case of *Fragaria x ananassa*) is the key factor in the differences in terms of aroma patterns between the two species. Yang et al. [20] evaluated the phenolic compounds present in *Fragaria x ananassa* Duch. cv. *Falandi* fruits, identifying flavone glucuronides, lignan glycosides, and other compounds. The authors also isolated, for the first time in the literature, three phenolic glucosides (2,3′′-epoxy-4-(butan-2-one-3-yl)-5,7,40-trihydroxy flavane 3-glucoside, kaempferol 3-(6-butylglucuronide), benzyl 2-glucosyl-6-rhamnosylbenzoate), offering their spectroscopic characteristics in support of the suggested structures. Roy et al. [21] studied comparatively the polyphenolic composition of *Fragaria x ananassa* and *Fragaria vesca* mutant fruits (white-colored) with regular fruits. Their study revealed the presence of 22 compounds belonging to different groups, as anthocyanins (cyanidin-3-glucoside, pelargonidin-3-glucoside, peonidin-3-glucoside, pelargonidin malonyl-glucoside, peonidin-malonyl-glucoside, and cyanidin-malonyl-glucoside) flavonols (quercetin, quercetin-3-glucoside, kaempferol-3-glucoside, kaempferol-acetyl glucoside, and kaempferol-coumaroyl hexoside), flavan-3-ols (proanthocyanidin dimers, catechin and epicatechin), hydroxycinnamic acids (caffeic acid, chlorogenic acid, and p-coumaroyl hexose), and ellagic acid-derived compounds (ellagic acid deoxyhexoside, methyl ellagic acid pentoside, and dimethyl ellagic acid pentoside). The major difference recorded between the white and red fruits (for both species) was the anthocyanins content. The white fruits had much lower total anthocyanin levels (0.11–0.35 for *F. vesca* and 0.89 mg/100 g fresh fruits for *Fragaria x ananassa*), compared with the red fruits (8.36, respectively 15.20 mg/100 g fresh fruits). Another major difference was recorded in terms of free ellagic acid and its derivatives (higher for *F. vesca* white fruits compared with the red fruits). Although the study was focused on the identification of specific mutations in different white-fruited genotypes, the article offers a very good insight on the variation of the phenolic composition, both with species and genotype. This could be further useful for the selection of the phenotype for separation of bioactive compounds for targeted applications. Two different compounds (a protease enzyme with molecular weight 65.8 kDa, stable at high temperatures and over a wide pH range, with specificity toward hemoglobin, respectively a cysteine protease inhibitor cystatin FchCYS1) were isolated in 2018 from *Fragaria x ananassa* [22], respectively *Fragaria chiloensis* [23], while a new ellagitannin (a galloylated derivative of agrimoniin, with molecular weight 2038) named fragariin A was isolated in 2019 by Karlińska et al. from strawberry fruits (*Fragaria x ananassa* Duch.) [24]. The distribution of the active compounds found in *Fragaria x ananassa* Duch. fruits was elucidated in 2019 by Nizioł et al. [25], by applying mass spectrometry imaging with ^109^Ag nanoparticle enhanced target. The authors studied thirty-two known metabolites and reached the conclusion that γ-aminobutyric acid, quinic acid, vitamin C, catechin, xylose, 4-hydroxy-2,5-dimethyl-3(2H)-furanone and nonanal are located under the fruit’s skin, aldehydes (hexanal, benzaldehyde) and ketones (1-penten-3-one, geranylacetone) are distributed throughout the fruits (in the inner core and in the cortex layer), while asparagine, lysine, gambriin C, oxalic acid and 2-methylbutanoic acid are found on/around the surface of the achenes. The authors suggested that their distribution is strongly connected with both the sites of their biosynthesis and to their function.

The composition of the fruits, although representing a characteristic of each species, can be influenced by a number of factors (as presented in Table 2), including the characteristics of the cultivar [21,27,29,31,34,39,43,44,45,46], cultivation factors, and the environmental conditions [18,27,37,38,47,48,49,50], ripening stage [28,29,35,51], or by biotechnological approaches [52,53,54]. For example, the variation in composition of a large number of *Fragaria × ananassa* Duch. cultivars was presented in 2019 by Nowicka et al. [43]. The authors identified as main components in the strawberry fruits the anthocyanins (pelargonidin-3-*O*-β-glucoside, cyanidin-3-*O*-β-glucoside, pelargonidin-3-*O*-rutinoside, cyaniding-3-*O*-(6″malonyl)glucoside, pelargonidin-3-*O*-(6″malonyl)glucoside)), flavonoids (derivatives of quercetin, kaempferol and isorhamnetin), cinnamic acid derivatives (isomers of 1-*O*-*p*-coumaroylhexose, 1-*O*-feruloylhexose, 1-*O*-*p*-coumaroyl-β-glucose, 1-*O*-trans-cinnamoyl-β-glucose), tannins and related compounds (gallotannins, glycosides of ellagic acid and methylellagic acid, free ellagic acid, flavan-3-ol derivatives), and triterpenoids (methyl or hydroxyl derivatives of tormentic or dihydrotormentic acid). More importantly, using the variation of the analyzed compounds allowed the authors to propose some of the cultivars for future studies and biomedical uses. Another interesting study was presented in 2011 by Pineli et al. who studied the compositional differences between two *Fragaria x ananassa* cultivars (*Osogrande* and *Camino Real*) in different ripening stages. Interestingly, the authors observed higher amounts of total phenolics, total ellagic acid, and vitamin C in the pink strawberries (3/4 ripe, as defined by the authors), compared with the green and fully ripe ones [28]. The differences recorded between the cited literature studies can be thus explained by the influence of those factors, as well as by the extraction procedure followed (which also have a strong influence on the final composition of the extracts), as demonstrated by Pawlaczyk-Graja et al. on the structure of polyphenolic-polysaccharide conjugates obtained from the leaves of *Fragaria vesca* using different classical and modern methods [55].

As the primary economic importance of the *Fragaria* genus is related to their fruits, it must be stated that, besides the previously presented factors, their composition (and finally, their health benefits) is correlated with the applied processing methods. The most encountered forms of products (besides fresh fruits) are represented by the dried fruits and puree. Méndez-Lagunas et al. [56] studied the influence of the thermal drying on the anthocyanins and total phenolic content of *Fragaria × ananassa* Duch. fruits. The study revealed a 26% loss of anthocyanins content when drying at 50 °C, respectively a 45% loss upon drying at 60 °C. The total phenolic content was even more seriously affected by the thermal process, with 60.9% loss associated with 50 °C, and 78.1% for the 60 °C treatment, respectively. Thus, the importance of fresh fruits consumption and when not possible, application of appropriate drying methods was emphasized; the authors propose the 50 °C treatment as suitable for preserving the bioactive compounds found in the strawberries (particularly anthocyanins). Álvarez-Fernández et al. [57] evaluated the variation in non-anthocyanin phenolic composition (hydrolyzed tannins, flavanols and condensed tannins, ellagic acid and derivatives, hydroxycinnamic acids, flavonol glycosides and stilbenes) of strawberry puree in different production stages. If mashing process affected the content of some phenolics (gallic acid, monogalloylglucoside and ellagic acid), the pasteurization process induced the decrease of all the compounds’ concentration. However, the non-anthocyanin phenolic profile was not significantly affected, suggesting that the strawberry puree represents a good source of phenolic compounds.

The strawberry “seeds” (achenes) were proven to be a valuable source of unsaturated fatty acids. Thus, several studies revealed that commercially available achenes oil contained high amounts of linoleic and α-linoleic acids (over 70%), with a total content of over 90% unsaturated fatty acids (also including oleic and traces of palmitoleic acid), while the saturated fatty acids were mainly represented by palmitic and stearic acids [58,59]. In the same time, the seeds could also be considered a source of dietary fibers, proteins, polyphenols (mainly ellagitannins), and vitamins [60]. Figure 1 summarizes the main classes of constituents found in *Fragaria* genus.

## 3. Biological Activities of *Fragaria* Genus

### 3.1. Antioxidant Properties

Traditionally consumed in the form of fruits (as previously presented), *Fragaria* species have also found application in traditional medicine. For example, *Fragaria vesca* leaves and fruits were traditionally used for the treatment of external rashes, as well as internally, as blood purification and roborontarium, for the treatment of diarrhea [61], as macerate for renal stones, or as tea (together with other medicinal plants) for treating stomach inflammations, sedation, or regulation of digestion [62]. The following paragraphs presents the main biological properties of different *Fragaria* species, as emerging from the literature data published in the past decade. Particularly, the anthocyanins family represent the subject of several review papers published in the last years, dealing with their bioavailability and potential health benefits [63,64,65]. The following chapters includes only the studies regarding the biological activity of compounds or extracts obtained from *Fragaria* species (not studies presenting the activity of compounds that are found in those plants).

The major classes of compounds found in the Fragaria species (anthocyanins and non-anthocyanin phenolic compounds) are known for their antioxidant properties [65,66]. As can be expected, the vast majority of the literature presenting the biological activities of species belonging to Fragaria genus present their antioxidant activity. However, the studies that will be presented should be carefully considered, as many are performed using assays predisposed to positive results in the presence of oxygenated functions on aromatic rings (thus being considered more “class-related” than as specific molecular targeting) [67]. Thus, those studies, although useful as screening tools, should be confirmed by more specific assays, such as in vivo or cell-based models [66,67].

Pineli et al. [28] performed a study on two Fragaria × ananassa Duch. cultivars (Osogrande and Camino Real), regarding the correlation between composition and the antioxidant activity (established using 2,2-Diphenyl-1-picrylhydrazyl radical-scavenging activity—DPPH and ferric reducing antioxidant power—FRAP assays) of acetone extracts obtained from fruits at different ripening stages. Very interestingly, although, as expected, the anthocyanins levels were higher in the red fruits (full ripe), the best antioxidant activities, for both cultivars and both assays were obtained for the pink fruits. The only exception is represented by the DPPH assay results for Osogrande cultivar (for which the best results were obtained for the red fruits); however, the differences between the results of the DPPH assay for the cultivar at the three ripening stages were not statistically significant. The differences between the results can be explained by the different mechanisms of the assays (as previously described by our group) [66]. A much better correlation was observed by the authors with the total phenolic and vitamin C content. Finally, the Osogrande cultivar presented superior antioxidant properties (associated with higher levels of the total phenolic and total ellagic acid content, especially in pink and red fruits). Zhu et al. [35] evaluated the influence of the solvent used for room-temperature extraction (water/ethanol) and of the extracted Fragaria × ananassa var. Amaou parts (red fruit, green fruit, red calyx, green calyx, flower, leaf, stolon, stolon leaf, stem, crown, and root) on the antioxidant properties of the extract. Their conclusion was that the ethanol was the solvent of choice (because of different polarities of the phenolic compounds found in the plant) and, among the plant parts, best results (presented as Trolox equivalents—TE per gram or extract and per 100 g of plant fresh weight—FW) were obtained for flower extracts (1460.1 μmol TE/g extract), respectively for crown (6212.3 μmol TE/100g FW), when reported to a fresh weight basis. Stolon leaves (1456.7 μmol TE/g extract, respectively 5244.2 μmol TE/100g FW) also exhibited a very good antioxidant activity.

Individual compounds (including phenolic glucosides, flavone glucuronides, and lignan glycosides) were isolated from the Fragaria x ananassa Duch. cv. Falandi fruits by Yang et al. [20] and tested for antioxidant properties. The best results were obtained for flavone glucuronides (in the ABTS and DPPH assays), and a lignan glycoside (in the FRAP assay), respectively. Considering the results obtained for the positive control used in the antioxidant assays (ascorbic acid), the authors suggested that the investigated phenolic compounds play an important role in the overall antioxidant property of the plant. Contrary to other studies, Chaves et al. [39] demonstrated a correlation between the total anthocyanin content and the antioxidant potential of strawberry fruits, in a study over seven cultivars. The antioxidant potential of plants, as it results from literature studies, seems to be correlated with total anthocyanins and not with total phenolic content.

In 2019, Nowicka et al. [43] published a study regarding the variation in composition and antioxidant properties of 90 cultivars of Fragaria × ananassa Duch. fruits over two years of production. The results (average values presented in Table 3) revealed not only that some cultivars can be considered as having superior antioxidant properties (Roxana, Gigaline, Selvik, Thuriga ISK, Eratina, Siria, Dagol, Plarionfre, Grenadier, and Kimberly), but also, considering the phytochemical profile, that the main compounds responsible for the activity are the tannins, especially ellagitannins and procyanidins.

As previously stated, because of the increasing request, strawberries are often commercialized as processed products. The effect of fruit drying on the antioxidant potential of the Fragaria × ananassa Duch., Diamante var. fruits was presented by Méndez-Lagunas et al. [56]. The antioxidant assay performed on the processed fruits (DPPH) revealed 74.7% loss of antioxidant activity for the thermal treatment at 50 °C, while the 60 °C treatment led to a 66.2% loss of the activity. The results suggested that, beyond temperature, heat treatment time has a stronger effect on the antioxidant activity (as at higher temperature, shorter periods are necessary). Similar, several researches were performed regarding the changes of antioxidant activity (determined using ORAC and DPPH assays) during different stages of puree production [57]. Although slight reduction of the antioxidant properties was recorded (statistically significant only for the pasteurization step), the authors recommended the strawberry puree as an excellent source of antioxidants. The same group [68] observed no effect of the gluconic fermentation of strawberry puree (applied for the production of beverages) on the antioxidant activity (determined using the DPPH assay); the authors even reported an increase of the antioxidant potential after the pasteurization step, which was correlated with an increase in the gallic acid and hydroxycinnamic derivatives content. The results would suggest that the gluconic fermentation could maintain the antioxidant potential of the fresh products upon processing.

The antioxidant potential of strawberries could rapidly find industrial applications, as was the case for other plant-derived antioxidants [69,70] in, for example, meat industry, as recently reviewed by Lorenzo et al. [71], for increasing the shelf-life of different products (as sausages or raw, cooked, and cooked-chilled porcine patties).

Table 3 summarizes the main findings regarding the antioxidant potential of Fragaria species, as well as the responsible classes of compounds (as presented by the authors).

As a general remark, it can be observed that most authors assign the antioxidant potential to the total phenolic content in general, and in particular to some classes of compounds, such as anthocyanins, flavan-3-ols or tannins. Considering the individual species, Fragaria x ananassa fruits presented antioxidant properties in the DPPH assay (the assays with the widest application) between 76.73–100 mg/mL (IC_50_) for various cultivars (the best results being obtained for the Camarosa cultivar) [39] or between 300 and 1300 μmol trolox/100 g fresh weight, for a larger survey (comprising 90 cultivars) [43]. Also, regarding the differences between the antioxidant potential of different plant parts, for F. chiloensis methanolic extracts the best activity was observed for fruits [26], while for Fragaria × ananassa for the crown ethanolic extract (6213.3 μmol trolox/100 g fresh weight). Fragaria vesca was mainly evaluated in terms of leaves, roots, or vegetative parts antioxidant activity, with antioxidant potential ranging from 13.46 mg/L to approx. 140 mg/L (IC_50_), strongly dependent on the source of vegetal material and applied extraction technique [72,73].

### 3.2. Anti-Inflammatory Properties

As previously stated, one of the traditional uses of *Fragaria* is as an *anti-inflammatory agent* [61,62]. Most of the authors assign the anti-inflammatory properties to the presence of anthocyanins (the most representative being pelargonidin and cyanidin derivates) [75], molecules with known anti-inflammatory potential [76,77], demonstrated both in vitro and in vivo [78,79]. Similar to the other biomedical potential, the anti-inflammatory action is also correlated with the composition of different *Fragaria* species. The traditional use of *F. vesca* as an anti-inflammatory agent was supported by the study of Liberal et al. [80]. The authors observed the decrease of a relevant mediator of the inflammatory response (nitric oxide) produced by macrophages, cultured in the presence of a NO-production inducing bacterial endotoxin (LPS). The ethanolic extract obtained from *Fragaria vesca* leaves, used at non-cytotoxic concentrations (80 and 160 mg/L), induced a 31%, and 40% inhibition, respectively. The authors assigned the NO decrease to a direct scavenging effect (as demonstrated by a 23% inhibition of the nitrite content in the culture media, correlated with the absence of a significant effect when quantifying the inducible nitric oxide synthase—iNOS and the pro-inflammatory cytokine IL-1β). The authors also observed a statistically insignificant increase in the phosphorylated IκBα (nuclear factor of kappa light polypeptide gene enhancer in B-cells inhibitor, alpha) content, suggesting either an increase of its expression or a decrease in its degradation. More than that, the authors observed an increased conversion of the microtubule-associated protein light chain LC3-I to LC3-II (a marker of autophagy), suggesting further anti-cancer properties. Methanolic extracts of *Fragaria x ananassa,* var. *Alba* fruits were also confirmed by Gasparrini et al. [81] to lower the intracellular levels of reactive oxygen species (ROS), decrease apoptotic rate and improve antioxidant defenses and mitochondria functionality in *E. Coli* induced inflammation in human dermal fibroblast cells. Their results showed significant decrease of TNF-α (tumor necrosis factor alpha), IL-1β and IL-6 (interleukin 6) levels. The authors proposed as responsible mechanism the action on AMPK (5′ AMP-activated protein kinase) related pathways (increment in phosphorylated AMPK expression). Between the two presented studies there are several experimental design/inputs differences that can explain the different obtained results (including the species and the solvent used for extraction). Thus, the higher levels of bioactive molecules in the *Fragaria x ananassa* fruits (compared with the *F. vesca* leaves, as previously presented) can explain the differences observed by the authors in terms of anti-inflammatory action. Similar observations were made by Molinett et al. [82] who proved the anti-inflammatory and hepatoprotective effect of aqueous *F. chiloensis* fruits extracts on LPS-inducted liver injury on rats. The anti-inflammatory effect of the *Fragaria x ananassa var. Camarosa* fruits was also in vivo evaluated on female mice by Duarte et al. [83], who assigned the activity to the presence of anthocyanins. Moreover, the authors performed in vitro experiments using pelargonidin-3-*O*-glucoside (the major anthocyanin in *Fragaria*) in order to establish its molecular mechanism of action. Regarding the in vivo experiments the authors noticed the inhibition of the carrageenan-induced leukocyte influx to the pleural cavity upon crude extract treatment, due to the reduction in neutrophil migration. The extract also induced a reduction of myeloperoxidase activity and reduced the exudate concentration in the pleural cavity and NO levels. The pure compound pelargonidin-3-*O*-glucoside produced similar results, inhibiting IkBα, also reducing the phosphorylation of p65 NF-kB (nuclear factor kappa-light-chain-enhancer of activated B cells) subunit. The authors proposed as mechanism of the anthocyanin the mitogen-activated protein kinase (MAPK) pathways, leading to the decrease in NF-kB and activated protein 1 (AP-1) translocation. In 2019, Van de Velde et al. [84] supported the previously reported anti-inflammation properties of strawberry extracts, also proposing another possible application, by evaluating the wound-healing effects. The extract and polyphenolics/anthocyanins-enriched fractions influenced the skin fibroblast migration (45% of the migration registered for the positive control—fetal bovine serum, for the crude extract, 50% of the positive control for the anthocyanins enriched fraction at 1 mg/L, 30% of the positive control for the polyphenolics enriched fraction), suggesting that the wound-healing properties are strongly associated with the anthocyanins presence. The dietary use of strawberry achenes commercial oil has also been proven to reduce the activity of superoxide dismutase (SOD) and glutathione peroxidase (cGPx) in rats (38.73, respectively 10.5 international units/gram of hemoglobin—U/g Hb, compared with the control group—67.33, and 22.9 U/g Hb, respectively), thus being qualified as a potential nutraceutical reducing oxidative stress [58].

### 3.3. Other Potential Applications

The *anti-microbial properties* were evaluated within the reviewed time period, especially for *F. vesca.* Hydromethanolic extracts obtained from leaves and roots of *Fragaria vesca* L. were evaluated by Gomes et al. [85] as antimicrobial agents a series of *S. aureus* strains. The results suggested a weak antimicrobial potential of the extracts (5–9 mm inhibition halos in the qualitative assays), which did not qualify the extracts for quantitative determinations. Superior results in terms of antimicrobial properties were obtained by Cardoso et al. [86]. Using hydroalcoholic extracts, the authors observed good antimicrobial properties of the crude extract against a series of *Helicobacter pylori* isolates (inhibition zones ≥ 15 mm) at a 25 mg/mL concentration. The ellagitannin-enriched fraction was efficient against all isolates at lower concentrations (7.5 mg/mL), which led the authors to assume that the ellagitannins were the main class of compounds responsible for the anti-microbial properties. As the *H. pylori* represents a pathogen involved in several gastric pathologies (including gastritis, gastroduodenal ulcer disease, gastric adenocarcinoma and mucosa-associated lymphoid tissue lymphoma), the authors proposed the wild strawberry extract as a potential candidate for human health applications.

The *anti-allergenic* potential of several compounds (linocinnamarin, 1-*O*-trans-cinnamoyl-b-d-glucopyranose, *p*-coumaric acid, cinnamic acid, chrysin, kaempferol, catechin, and trans-tiliroside) isolated from *Fragaria x ananassa* var. *Minomusume* fruits were evaluated by Ninomiya et al. [87], through the determination of their inhibitory effects on antigen-stimulated degranulation in rat basophilic leukemia RBL-2H3 cells. Among the studied compounds, linocinnamarin (95% inhibition of control at 100 μM) and cinnamic acid (approx. 80% of control at 100 μM) were the most efficient in degranulation suppression (through direct inactivation of spleen tyrosine kinase), being proposed as promising tools for alleviating symptoms of type I allergy.

The commercially-available strawberry freeze-dried powder was demonstrated by Abdulazeez [88] to reverse alloxan-induced diabetes (results not presented in Table 4 as authors used commercial powder product); in a similar study, Yang et al. [12] evaluated the potential *anti-diabetic application* of new and known compounds isolated from strawberry fruits (as presented in Section 2) by determining the α-glucosidase inhibitory activity. The best results were obtained for cupressoside A (IC_50_ = 25.39 μM), kaempferol 3-(6-methylglucuronide) (IC_50_ = 65.22 μM), and 2-*p*-hydroxybenzoyl-2,4,6-*tri* hydroxyphenylacetate (IC_50_ = 97.81 μM), with very good results obtained for a newly proposed structure (kaempferol 3-(6-butylglucuronide)-IC_50_ = 107.52 μM); results superior to the positive control (acarbose-IC_50_ = 619.94 μM) were also obtained for five other compounds.

Another interesting study is represented by the one performed by Zhu et al. [35]. Besides the phytochemical and anti-oxidant studies (previously presented), the authors also evaluated the anti-obesity, anti-allergy, and skin-lightening effects of extracts obtained from different parts of strawberry in different ripening stages. The extracts exhibited anti-obesity activity (the water extract of unripe fruit and the ethanol extracts of the stem, stolon leaf, and crown ripe fruits exhibiting anti-lipase activity, as well as inhibitory effect on adipocyte differentiation), anti-allergy function (the ethanol extracts of flower, stolon leaf and red calyx showing strong suppression effect on the release of β-hexosaminidase), and skin-lightening potential (ethanol extracts of ripe fruits, unripe fruit and the crown exhibiting melanogenesis inhibitory action, correlated with the tyrosinase-inhibitory activities).

The *cytotoxic potential* of the *Fragaria* species was demonstrated in several studies. Somasagara et al. [89] evaluated the potential application of methanolic strawberry extract in leukemia (CEM) and breast cancer (T47D) cell lines ex vivo, as well as its therapeutic and chemopreventive potential in vivo. The MTT, trypan blue and LDH assays revealed the cytotoxicity of the extract on cancer cells, in a concentration-dependent manner, while the in vivo studies revealed the anti-proliferative action on tumor cells. Forni et al. [90] investigated the antiproliferative and differentiation potential of an anthocyanin-rich strawberry fruit extract on B16-F10 murine melanoma cells. Their results showed the reduction of cell proliferation (30% after 48 h), accompanied by the lowering of the intracellular levels of polyamines (63.8% decrease of spermidine, 52.9% decrease of spermine, after 72 h), and the enhancement of tissue transglutaminase (172% increase after 48 h). The used extract also down-regulated p53 and p21 expression (47.2%, and 32.6%, respectively). Liberal et al. [33] presented the cytotoxic potential of an ellagitannin-enriched fraction from *Fragaria vesca* leaves on human hepatic carcinoma cell line (HepG2). Their results showed that the crude extract and, more pronounced, the ellagitannin-enriched fraction, were able to interfere with cell cycle distribution. The ellagitannin-enriched fraction also induced necrosis and apoptosis in the threated cells, decreased chymotrypsin-like activity of the 26S proteasome, impaired autophagic flux, promoted the accumulation of ubiquitinated proteins, and decreased the expression of several proteasome subunits. Lucioli et al. [91] evaluated the influence of hydroalcoholic extracts (methanol, ethanol, isopropanol) from in vitro cell suspension on the proliferation of several cancer cells (neuroblastoma, colon, and cervix carcinoma cell lines). The extracts induced a statistically significant reduction of cell growth but did not affect the human fibroblasts from healthy donors. The chemoprotective action of strawberries was also studied by Casto et al. [92] (results not presented in Table 4 as authors used commercial strawberry powder). The chemoprotective role of strawberries on colorectal cancer in inflammatory bowel disease was recently reviewed by Chen et al. [93], who proposed a mechanism of action involving the suppression of cytokines release, decrease of oxidative stress, reduction of genomic instability, and inhibition of NFκB (nuclear factor kappa-light-chain-enhancer of activated B cells) and related signalling pathways. Table 4 summarizes the main biological activities (except anti-oxidant properties, presented in Table 3), as emerging from the literature survey, considering the main constituents of the tested extracts.

## 4. Current Limitations and Future Perspectives

In spite of the wide spread of the *Fragaria* genus, few species represent the subject of the last decade scientific research, with many works focused on the composition and bioactivities of wild and garden species. Although those species, with certain commercial value, represent a very valuable source of different classes of polyphenols (including proanthocyanidins, anthocyanins, ellagitannins, flavonoids, phenylpropanoids, stilbenes, phenol glycosides, and dihydrochalcones) and thus possessing important nutritional value [96], the researchers should also focus on less-studied species, native to different parts of the world. This could represent an important opportunity for future studies. Also, the evaluation of other possible application (such as cosmetic products) could represent an interesting area of research. As an example, Sikora et al. [59] used supercritical CO_2_ extraction for obtaining strawberry seeds (achenes) oil and applied it (in varying concentration from 0.5–2%) for the development of shower/bath cosmetics with good skin-moisturizing properties, without influencing the stability of the products.

A drawback that limits the potential beneficial effects of the strawberries’ consumption is represented by their processing. As previously mentioned, the thermal treatment reduces both the bioactive compounds and their biological activities. In this area, new protective coatings obtained using nanotechnological approaches were proposed for increasing their shelf-lives [97], or as post-harvest treatments, alternatives to the classical thermal treatments currently applied [98,99].

As emerging from the literature study, most of the research is performed via classical extraction methods in order to align with traditional uses of the species (infusion, decoction), and only few are including modern extraction and separation techniques (such as countercurrent chromatography). In this area, would be beneficial to evaluate the use of modern methods of extraction/separation of biological active compounds [100].

The rich anthocyanin content of the genus seems to offer promising compounds for very important applications, such as anti-cancer or chemoprotective agents. Another surprising aspect emerging from the reviewed works is the relatively few studies concerning the potential of *Fragaria* species towards anti-microbial applications. Although the literature abounds in examples regarding the anti-microbial potential of different plants extracts, it appears that the *Fragaria* could still offer some surprising results in this domain (considering its rich composition). Also, the application of *Fragaria* extracts in other areas, such as nanotechnology, is only “surface-scratched” at this moment. For example, Demirbas et al. [101] evaluated anthocyanins-rich berry extracts (including strawberry) for the phytosynthesis of silver nanoparticles and evaluated their antioxidant and anti-microbial potential. Although the results of the phytosynthesis process were promising (smallest dimensions, compared with the blackberry or raspberry extracts (35 nm), and with less aggregation, the antimicrobial effect was relatively poor (and only on *B. cereus*). The field of nanotechnology in general, and of nanoparticles phytosynthesis in special, represents a continuously increasing domain, so the extracts obtained from different species of the genus could demonstrate their usefulness in this area [102]. Other different nanoparticles could be obtained using the extracts, as well with tuned properties, using enrichment of the extracts [103]. Correlated with the anti-microbial properties, the potential toward nanotechnology could also lead to the development of other materials (such as polymeric encapsulated nanoparticles or even natural extracts) for different anti-microbial applications or for wider applications in increasing the quality of food products [104,105].

Finally, as a more general remark, the absence of standardized methods for the evaluation of different potential applications represents a major draw-back in the comparison of the results presented by different authors.

## 5. Conclusions

*Fragaria* represents a widely spread genus, with species encountered all over the world. The current study aimed to present the progress made in the last decade in the study of the composition and potential applications of the species belonging to the *Fragaria* genus. However, in spite the wide spread of its species, only a few represents the subject of current research. The literature study revealed that three species represent the major subject of research, respectively the wild, garden and beach strawberry.

Used in traditional medicine especially as an anti-inflammatory adjuvant, the scientific research supports this application, as well as several other potentially important uses, for example as a chemoprotective agent.

The composition of the genus, rich in polyphenolic compounds in general, and in anthocyanins in particular, suggests its possible application in multiple other areas. The relatively under-study of the genus (and the severe lack of literature for some of the species) offers in turn an opportunity for future research. At the same time, elucidation of the composition and properties of the commercially valuable products represents a very important aspect, as the characteristics of such a widely consumed product should be thoroughly elucidated.

## Figures and Tables

**Table 1:** Major (common) components in *Fragaria* L. aggregate fruits (adapted from [11,12,13,14,15]).

Class	Compound	Ref.
Anthocyanins	Pelargonidin 3-glucoside, cyanidin 3-glucoside, cyanidin 3-rutinoside, pelargonidin 3-galactoside, pelargonidin 3-rutinoside, pelargonidin 3-arabinoside, pelargonidin 3-malylglucoside	[11,12,13]
Flavonols	Quercetin, kaempferol, fisetin, their glucuronides, and glycosides	[11,12,13,16]
Flavanols	Catechin, proanthocyanidin B1, proanthocyanidin trimer, proanthocyanidin B3	[11]
Ellagitannins	Sanguiin H-6, ellagitannin, ellagic acid, lambertianin C, galloylbis-hexahydroxydiphenoyl-glucose	[11]
Phenolic acids	4-coumaric acid, p-hydroxybenzoic acid, ferulic acid, vanillic acid, sinapic acid	[15]
Vitamins	Vitamin C, vitamin B9	[14]
Minerals	Mn, K, Mg, P, Ca	[11]
Others	Sugars (glucose, fructose, and sucrose), fibers	[11]

**Table 2:** Composition of *Fragaria* species (as presented by original works published in the reviewed period; references presented in chronological order).

Species	Plant Part, Other Variables	Identified Compounds and Main Findings	Identification Method	Ref.
*F. chiloensis*	Ripe fruits	Anthocyanins (cyanidin 3-*O*-glucoside, pelargonidin 3-*O*-glucoside cyanidin-malonyl-glucoside and pelargonidin-malonyl-glucoside); procyanidins, ellagitannins, ellagic acid and flavonol derivatives	HPLC-DAD, LC-ESI-MS	[26]
*F. chiloensis*	Leaves	Procyanidins, ellagitannins, ellagic acid and flavonol derivatives	HPLC-DAD, LC-ESI-MS	[26]
*F. chiloensis*	Rhizomes	Procyanidins, ellagitannins, ellagic acid and flavonol derivatives	HPLC-DAD, LC-ESI-MS	[26]
*Fragaria × ananassa*	Fruits	Anthocyanins (pelargonidin-3-glucoside, pelargonidin-3-rutinoside, cyanidin-3-rutinoside, pelargonidin-3,5-diglucoside, pelargonidin-3-(6-acetyl)-glucoside, 5-carboxypyranopelargonidin-3-glucoside, delphinidin-3-glucoside, peonidin-3-glucoside, cyanidin-3-galactoside), *p*-hydroxybenzoic acid, (+)-catechin, ellagic acid, *p*-coumaric acid, quercetin glucoside	LC-MS/MS, HPLC-UV/Vis	[17]
*Fragaria × ananassa*	Fruits, cultivar and seasonal variations	Vitamin C, β-carotene, total phenolics, total anthocyanins; genotype influence is stronger than the environmental influence	Colorimetric	[27]
*Fragaria × ananassa*	Fruits, different cultivars on different ripeness stage	Total vitamin C, total phenolics, total anthocyanins, total ellagic acid/pelargonidin-3-glucoside and cyanidin-3-glucoside; higher amounts in pink fruits compared with fully ripped fruits	Colorimetric/HPLC-DAD	[28]
*Fragaria × ananassa*	Fruits, different farming methods	Total phenolics/pelargonidin-3-glucoside and cyanidin-3-glucoside, vitamin C, higher in organic farming fruits	Colorimetric/HPLC-DAD	[18]
*Fragaria × ananassa*	Fruits, different cultivars (27) and ripening stages	Phenolic compounds (multiple classes, including anthocyanins, flavanols and ellagitannins); composition dependent on cultivar, cinnamic acid conjugates and anthocyanins levels increased with the ripening stage	HPLC-DAD-MS	[29]
*Fragaria × ananassa*, *F. vesca*	Fruits	Quercetin and isorhamnetin glycosides (higher levels in wild strawberry)	HPLC-DAD, LC-ESI-MS	[30]
*Fragaria × ananassa*, *F. vesca*	Fruits, different cultivars	Volatile esters (including ethyl acetate, hexyl acetate, methyl butanoate, ethyl butanoate, hexyl butanoate, methyl hexanoate, ethyl hexanoate, hexyl hexanoate); higher levels in cultivated strawberries.	GC-MS	[19]
*F. vesca*	Fruits, two different cultivars	Anthocyanins (cyanidin 3-*O*-glucoside, pelargonidin 3-*O*-glucoside, peonidin 3-*O*-glucoside, cyanidin 3-*O*-malonylglucoside, pelargonidin 3-*O*-malonylglucoside, peonidin 3-*O*-malonylglucoside), dihydroflavonol and flavonols (taxifolin 3-*O*-arabinoside, kaempferol 3-*O*-glucoside, quercetin 3-*O*-glucoside, quercetin-acetylhexoside, kaempferol 3-*O*-acetylhexosides), flavan-3-ols and proanthocyanidins (catechin, B type proanthocyanidin dimers, trimers, and tetramers), ellagic acid and derivatives (glycosylated, methyl pentoside, methylellagic acid methyl pentoside, ellagitannins), other compounds (benzoic acid, ferulic acid hexose derivative, citric acid, furaneol glucoside)	HPLC-DAD	[31]
*Fragaria × ananassa*, *F. vesca*	Fruits	Anthocyanins (cyanidin, pelargonidin), cyanidin glycosides (cyanidin 3-glucoside, cyanidin 3-arabinoside, cyanidin 3-sambubioside, delphinidin 3-galactoside, delphinidin 3-glucoside, delphinidin 3-malonylglucoside); higher levels of cyanidin glycosides in wild species	HPLC-DAD	[32]
*F. vesca*	Leaves	Ellagitannins (sanguiin H-2 isomer, sanguiin H-10 isomer, sanguiin H-6/agrimoniin/lambertianin A isomer, castalagin/vescalagin isomer, sanguiin H-10 isomer, sanguiin H-2 isomer, casuarictin/potentillin isomer)	LC-PDA-ESI-MS	[33]
*Fragaria × ananassa*	Fruits, different cultivars and production years	Vitamin C, anthocyanins (pelargonidin 3-glucoside, cyanidin 3-glucoside, pelargonidin 3-rutinoside), ellagic acid; strongly dependent on the cultivar and production year	HPLC-UV/Vis	[34]
*Fragaria × ananassa*	Fruits, at different ripening stage	Vitamin C, pelargonidin-3-rutinoside, ellagic acid, cyanidin-3-glucoside, quercetin (red fruits), neochlorogenic, pelargonidin-3-glucoside, pelargonidin-3-rutinoside, epicatechin, quercetin-3-β-d-glucoside, ellagic acid (green fruits)	LC-ESI-TOF	[35]
*Fragaria × ananassa*	Calyx (red and green)	Quercetin-3-β-d-glucoside, ellagic acid, kaempferol-3-*O*-glucoside, vitamin C (red), catechin, quercetin-3-β-d-glucoside, ellagic acid (green)	LC-ESI-TOF	[35]
*Fragaria × ananassa*	Flower	Catechin, quercetin-3-β-d-glucoside, ellagic acid, kaempferol-3-*O*-glucoside, vitamin C	LC-ESI-TOF	[35]
*Fragaria × ananassa*	Leaf	Procyanidin dimer and trimer, catechin, quercetin-3-β-d-glucoside, vitamin C, ellagic acid	LC-ESI-TOF	[35]
*Fragaria × ananassa*	Stolon	Neochlorogenic, procyanidin dimer, catechin, quercetin-3-β-d-glucoside, ellagic acid, vitamin C, kaempferol-3-*O*-glucoside	LC-ESI-TOF	[35]
*Fragaria × ananassa*	Stem	Procyanidin dimer, catechin, ferulic acid, quercetin-3-β-d-glucoside, ellagic acid	LC-ESI-TOF	[35]
*Fragaria × ananassa*	Crown	Procyanidin dimer and trimer, catechin, propelargonidin dimer, ellagic acid	LC-ESI-TOF	[35]
*Fragaria × ananassa*	Root	Procyanidin dimer and trimer, catechin, neochlorogenic, propelargonidin dimer	LC-ESI-TOF	[35]
*Fragaria × ananassa*	Fruits, different novel cultivars	Phenolic acids (p-coumaric acid, ellagic acid, ferulic acid derivative, *p*-coumaric acid derivatives), monomeric flavanols ((+)-catechin), flavonols (quercetin 3-*O*-glucoside, fisetin, quercetin 3-*O*-glucoside derivative), anthocyanins (cyanidin 3-glucoside, cyanidin 3-rutinoside, cyanidin pentoside, pelargonidin 3-galactoside, pelargonidin 3,5-diglucoside, pelargonidin 3-glucoside, pelargonidin 3-rutinoside, cyanidin 3-Oacetylglucoside, cyanidin hexoside, pelargonidin 3-*O*-monoglucuronide, pelargonidin derivatives)	HPLC-DAD, LC-ESI-QTOF	[36]
*Fragaria × ananassa*	Fruits, grown on different altitudes, on consecutive years	Hydroxybenzoic acid, *p*-coumaric acid, other hydroxycinnamic acids, (+)-catechin, (−)-epicatechin, procyanidins, flavonols, anthocyanins (cyanidin 3-glucoside, pelargonidin 3-glucoside, pelargonidin derivative); higher levels recorded at lower altitudes.	HPLC-DAD	[37]
*Fragaria × ananassa*	Fruits	Kaempferol 3-(6-methylglucuronide), quercetin 3-(6-methylglucuronide), isorhamnetin 3-(6-methylglucuronide), trichocarpin, 2-*p*-hydroxybenzoyl-2,4,6-tri hydroxyphenylacetate, 2-*p*-hydroxyphene thyl-6-caffeoylglucoside, zingerone 4-glucoside, b-hydroxypropiovanillone 3-glucoside, (+)-isolariciresinol 90-glucoside, (−)-isolariciresinol 90-glucoside, aviculin, (−)-secoisolariciresinol 4-glucoside, cupressoside A, cedrusin, icariside E4, dihydrodehydrodiconiferyl alcohol 90-glucoside, massonianoside A, urolignoside, (−)-pinoresinol 4-glucoside, 2,3”-epoxy-4-(butan-2-one-3-yl)-5,7,40-trihydroxy flavane 3-glucoside, kaempferol 3-(6-butylglucuronide), benzyl 2-glucosyl-6-rhamnosylbenzoate	^1^H NMR, ^13^C NMR, HMBC, HPLC-UV/Vis, LC-MS/MS, HR-ESI-MS,	[20]
*F. vesca*	Fruits, wild and cultivated, from different geographical areas	39 phenolic compounds (including cyanidin 3-*O*-glucoside, delphinidin-3-*O*-glucoside, pelargonidin-3-*O*-glucoside, pelargonidin-3-*O*-rutinoside, (+) catechin, (−) epicatechin, procyanidin B1 and B2, isoquercetin, gallic acid, *p*-coumaric acid, phloridzin); composition dependent on the geographical area	LC-ESI-Orbitrap-MS, LC-ESI-QTrap-MS, LC-ESI-QTrap-MS/MS	[38]
*Fragaria × ananassa*	Fruits, different cultivars	Cyanidin 3-*O*-glucoside, pelargonidin-3-*O*-glucoside, pelargonidin-*O*-rutinoside, total anthocyanins content, dependent on the cultivar	UPLC-PDA-ESI-MS, HPLC-DAD	[39]
*F. vesca*	Fruits	Volatile composition—one hundred compounds (including esters, aldehydes, ketones, alcohols, terpenoids, furans and lactones).	GS-MS	[40]
*F. vesca*	Leaves	27 metabolites (organic acids, flavonoids, catechin and its oligomers, ellagitannins), including quinic acid, chelidonic acid, quercetin derivatives, catechin and procyanidins, phloridzin, pedunculagin, methyl ellagic acid glucuronide.	LC-ESI-Orbitrap-MS	[41]
*Fragaria × ananassa*, *F. vesca*	White-fruited mutants, different genotypes	Anthocyanins, flavonols, flavan-3-ols, hydroxycinnamic acids, and ellagic acid—derived compounds, dependent on genotype	LC-ESI-MS/MS	[21]
*F. chiloensis*	Fruits	Anthocyanins (cyanidin-3-*O*-glucoside, pelargonidin hexoside, cyanidin manlonyl hexoside, pelargonidin-malonyl hexoside), ellagitannins (ellagic acid hexoside, pentoside, rhamnoside), proanthocyanidin dimers, epicatechin, flavonols (quercetin pentoside, glucuronide)	HPLC-DAD, LC-ESI-MS	[42]
*Fragaria × ananassa*	Fruits, different cultivars	Anthocyanins, flavonoids, cinnamic acid derivatives, tannins and related compounds, triterpenoids; concentration dependent on the cultivar	UPLC-ESI-QTOF-MS/MS, HPLC-DAD	[43]

*where:*^13^C NMR—Carbon-13 nuclear magnetic resonance; GC-MS—gas chromatography–mass spectrometry; ^1^H NMR—proton nuclear magnetic resonance; HMBC —heteronuclear multiple bond correlation; HPLC-DAD—high-performance liquid chromatography with diode array detector; HPLC-UV/Vis—high-performance liquid chromatography equipped with UV/vis detector; HR-ESI-MS—high-resolution electrospray ionization mass spectrometry analysis; LC-ESI-MS(/MS)—liquid chromatography electrospray ionization (tandem) mass spectrometry analysis; LC-ESI-Orbitrap-MS—liquid chromatography electrospray ionization Orbitrap mass spectrometry; LC-ESI-QTrap-MS(/MS)—liquid chromatography electrospray ionization quadrupole ion trap mass spectrometry; LC–ESI–(Q)TOF—liquid chromatography electrospray ionization with (quadrupole) time-of-flight; LC-MS/MS—liquid chromatography–tandem mass spectrometry; LC-PDA-ESI-MS—liquid chromatography equipped with photodiode array detector coupled to mass spectrometry using the electrospray ionization interface; UPLC-ESI-QTOF-MS/MS—ultra-performance liquid chromatography equipped quadrupole time of flight coupled to tandem mass spectrometry using the electrospray ionization interface; UPLC-PDA-ESI-MS—ultra-performance liquid chromatography equipped with photodiode array detector coupled to mass spectrometry using the electrospray ionization interface.

**Table 3:** Antioxidant properties of different extracts obtained from *Fragaria* species (references presented in chronological order).

Species	Extraction Method	Antioxidant Assay	Antioxidant Potential	Responsible Compounds	Ref.
*Fragaria × ananassa*, *Camarosa* var. fruits	Anthocyanins isolated using CCC	ORAC, FRAP	ORAC: 2.7–24.46 mmol Trolox/g; FRAP: 2.75–12.5 mmol Fe^2+^/g (depending on the fraction)	Anthocyanins	[17]
*Fragaria chiloensis* spp. *chiloensis* form *chiloensis* fruits	Methanol: formic acid (99:1 *v*/*v*) extraction	DPPH, SAS	DPPH assay: IC_50_ = 38.7 mg/L; SAS: 79.3%)	Aglycone and glycosylated ellagic acid and flavonoids	[26]
*Fragaria chiloensis* spp. *chiloensis* form *chiloensis* leaves	Methanol: formic acid (99:1 *v*/*v*) extraction	DPPH, SAS	DPPH assay: IC_50_ = 49.4 mg/L; SAS: 67.60%	Aglycone and glycosylated ellagic acid and flavonoids	[26]
*Fragaria chiloensis* spp. *chiloensis* form *chiloensis* rhizomes	Methanol: formic acid (99:1 *v*/*v*) extraction	DPPH, SAS	DPPH assay: IC_50_ = 64.8 mg/L; SAS: 55%	Aglycone and glycosylated ellagic acid and flavonoids	[26]
*Fragaria x ananassa Osogrande* var. frozen fruits	Acetone (80%) extraction	DPPH, FRAP	DPPH: 11.91–12.83 μmol BHT eq./g FW; best results for ripe fruits FRAP: 27.37–36.75 μmol FS eq./g FW; best results for green fruits	Total phenolic content, vitamin C	[28]
*Fragaria x ananassa Camino Real* var. frozen fruits	Acetone (80%) extraction	DPPH, FRAP	DPPH: 9.75–12.01 μmol BHT eq./g FW, FRAP: 24.13–28.49 μmol FS eq./g FW (best results for pink fruits)	Total phenolic content, vitamin C	[28]
*F. vesca* leaves	Methanol, ultrasounds extraction	DPPH, FRAP	DPPH: IC_50_ = 13.46 mg/L; FRAP: 0.878 mmol Fe^2+^/g DW	Total phenols, total tannins	[72]
*F. vesca* roots, wild-growing	Hydromethanolic extraction, infusion, decoction	DPPH, FRAP, β-Carotene bleaching inhibition, TBARS	IC_50_, mg/L: DPPH—50.03/50.56/50.62; FRAP—40.98/44.78/49.23; β-C bleaching—116.26/44.88/66.10; TBARS—35.76/4.76/6.14	Total phenolics, total flavan-3-ols, total dihydroflavonols,	[73]
*F. vesca* roots, commercial	Hydromethanolic extraction, infusion, decoction	DPPH, FRAP, β-Carotene bleaching inhibition, TBARS	IC_50_, mg/L: DPPH—68.89/255.81/51.32; FRAP—327.75/78.99/67.92; β-C bleaching—68.34/23.44/114.67; TBARS—6.69/24.25/10.62	Total phenolics, total flavan-3-ols, total dihydroflavonols,	[73]
*Fragaria × ananassa* var. *Amaou,* fruits, at different ripening stage	Ethanol or water room temperature extraction	Modified ABTS assay	Ethanol: 150.5/151.9; water: 227.2/189.4 (red/green fruits) μmol TE/100 g FW	Total phenolic content	[35]
*Fragaria × ananassa* var. *Amaou* calyx (red and green)	Ethanol or water room temperature extraction	Modified ABTS assay	Ethanol: 241.1/1239.9; water: 1716.6/577.7 μmol TE/100 g FW (red/green calyx)	Total phenolic content	[35]
*Fragaria × ananassa* var. *Amaou* flower	Ethanol or water room temperature extraction	Modified ABTS assay	4234.4/387.5 μmol TE/100 g FW (ethanol/water)	Total phenolic content	[35]
*Fragaria × ananassa* var. *Amaou* leaves	Ethanol or water room temperature extraction	Modified ABTS assay	2401.7/241.1 μmol TE/100 g FW (ethanol/water)	Total phenolic content	[35]
*Fragaria × ananassa* var. *Amaou* stolon	Ethanol or water room temperature extraction	Modified ABTS assay	1089.4/1856.7 μmol TE/100 g FW (ethanol/water)	Total phenolic content	[35]
*Fragaria × ananassa* var. *Amaou* stem	Ethanol or water room temperature extraction	Modified ABTS assay	1338.6/1123.1 μmol TE/100 g FW (ethanol/water)	Total phenolic content	[35]
*Fragaria × ananassa* var. *Amaou* crown	Ethanol or water room temperature extraction	Modified ABTS assay	6213.3/128.7 μmol TE/100 g FW (ethanol/water)	Total phenolic content	[35]
*Fragaria × ananassa* var. *Amaou* root	Ethanol or water room temperature extraction	Modified ABTS assay	253.1/69.2 μmol TE/100 g FW (ethanol/water)	Total phenolic content	[35]
*F. vesca* vegetative parts (leaves and stems), wild-growing	Hydromethanolic and aqueous extracts; wild-growing infusion microencapsulated in alginate and incorporated in k-carrageenan gelatine	DPPH, FRAP, β-Carotene bleaching inhibition, TBARS	IC_50_, mg/L: DPPH—123.67/86.17/109.10; FRAP—81.40/62.36/77.28; β-C bleaching—56.71/12.34/13.40; TBARS—12.63/3.12/5.03 (hydromethanolic/infusion/decoction); Final formulation (mg/mL)—DPPH—2.74; FRAP = 1.23	Total phenolics, total flavan-3-ols, total dihydroflavonols,	[74]
*F. vesca* vegetative parts (leaves and stems), commercial	Hydromethanolic and aqueous extracts	DPPH, FRAP, β-Carotene bleaching inhibition, TBARS	IC_50_, mg/L: DPPH—139.33/121.94/118.89; FRAP—324.49/91.88/88.20; β-C bleaching—388.90/76.41/69.98; TBARS—24.36/23.07/17.52 (hydromethanolic/infusion/decoction).	Total phenolics, total flavan-3-ols, total dihydroflavonols,	[74]
*Fragaria x ananassa* cv. *Falandi* fruit	22 compounds isolated from ethanolic extracts	ABTS, DPPH, FRAP	Best results (IC_50_): ABTS—4.42 μM kaempferol 3-(6-methylglucuronide); DPPH—32.12 μM quercetin 3-(6-methylglucuronide); FRAP—0.05 mmol/g—urolignoside.	Individual compounds	[20]
*Fragaria x ananassa* cv. *Albion, Aromas, Camarosa, Camino Real, Monte Rey, Portola*, and *San Andreas* fruits	Ultrasonic extraction with acidified methanol	DPPH	IC_50_ (mg/mL) ranging from 76.73 (*Camarosa*)—100 (*Camino Real*)	Total anthocyanin content	[39]
*F. vesca* leaves native to Italy	Ultrasonic extraction with ethanol: water solvent (70:30, *v*/*v*)	TEAC	0.34–0.35 mg/mL Trolox eq., compared with quercetin (0.40)	Condensed tannins and flavonoid derivatives	[41]
*Fragaria x ananassa* cv. *Tochiotome* leaves	Supercritical CO_2_ extraction with different entrainers	DPPH	0.07 (simple supercritical extraction)—5.82 μmol BHT/g sample (with ethanol, dried at 40 °C)	Phenolic compounds	[10]
*Fragaria × ananassa* fruits (90 cultivars)	Ultrasonic aqueous methanol (70%) acidified with 1.5% formic acid, at room temperature	DPPH, ABTS	Average values (μmol Trolox/100 g):765.06 (DPPH), 1637.96 (ABTS)	Tannin-based compounds.	[43]

*where:* ABTS—2,2′-azino-bis(3-ethylbenzothiazoline-6-sulfonic acid) assay; BHT—butylated hydroxytoluene; DPPH—reduction of 2,2-diphenyl-1-picrylhydrazyl; DW—dry weight; eq.—equivalents; FRAP—ferric reducing ability of plasma; FS—ferrous sulphate; FW—fresh weight; IC_50_—half maximal inhibitory concentration; ORAC—oxygen radical absorbance capacity; SAS—superoxide anion assay; TBARS—thiobarbituric acid reactive substances assay; TEAC—Trolox equivalent antioxidant capacity.

**Table 4:** Main biological activities presented in the literature (references listed in chronological order).

Action	Plant	Extraction Method	Assay	Results	Responsible Compounds	Ref.
Anti-inflammatory on inflammatory bowel disease	*Fragaria vesca* leaves	Eth. extraction	MPO activity; GSH, SOD and CAT levels	Prevention of increase in colon weight and disease activity index, decrease in macroscopic and microscopic lesion score; significant improvement of MPO, CAT and SOD levels at 500 mg/kg 5 days oral treatment	Phenolic acids, flavonoids	[94]
Anti-inflammatory	*Fragaria vesca* leaves	Eth. extraction at room temperature, infusion	Nitric oxide production, western blot analysis (expression of pro-inflammatory proteins in lipopolysaccharide-triggered macrophages); nitric oxide scavenger activity	Inhibition of nitrite production on pre-treated cells (at 80 and 160 mg/L—31%/40%); 23% inhibition in culture media, at 160 mg/L	Phenolic content	[80]
Anti-inflammatory	*Fragaria x ananassa,* var. *Alba* fruits	Meth. extraction at room temperature, infusion	Determination of ROS intracellular levels, apoptosis detection, antioxidant enzyme activities, immunoblotting analysis, determination of mitochondrial respiration and extracellular acidification rate in cells	Reduction of intracellular ROS levels (significant at 100 mg/L), decreased apoptotic rate (significant at 50 and 100 mg/L); Increased ARE-antioxidant enzymes expression, reduced NO and inflammatory cytokines production (at 50 and 100 mg/L) to control levels	Vitamin C, anthocyanins, flavonoids	[81]
Anti-inflammatory, hepatoprotective	*Fragaria chiloensis*ssp. *Chiloensis* fruits	Aq. extracts	Histological analyses, determination of transaminases, cytokines, F2-isoprostanes, and glutathione assays	maintained hepatocellular membrane, structural integrity, attenuated hepatic oxidative stress, and inhibited inflammatory response in LPS-induced liver injury; downregulation of cytokines (TNFa, IL-1β, and IL-6)	Phenolic content	[82]
Anti-inflammatory	*Fragaria x ananassa* var. *Camarosa* fruits	Ultrasonic-assisted, acidified meth. extraction, separation	*In vivo:* quantification of the leukocyte content, exudate concentration, MPO and ADA activities, nitric oxide products, TNF-α and IL-6 levels; in vitro: MTT assay, measurement of nitric oxide products, TNF-α and IL-6 levels, western blot analysis	Inhibition of the carrageenan-induced leukocyte influx to the pleural cavity; reduction of myeloperoxidase activity, exudate concentration, NO levels.	Phenolic compounds, anthocyanins (particularly pelargonidin-3-*O*-glucoside)	[83]
Anti-inflammatory, wound healing	*Fragaria x ananassa* var. *San Andreas* fruits	Ultrasound-assisted extraction, acidified meth.: aq. (80:20); separation of different fractions	MTT assay, ROS, NO levels, effects on inflammatory markers and on skin fibroblast migration	ROS reduction, suppression of IL-1β, IL-6 and iNOS gene expressions; enhanced skin fibroblast migration	Polyphenolic compounds, especially anthocyanins	[84]
Anti-microbial	*Fragaria vesca* leaves and roots	Centrifugation extraction with meth.: aq. (80:20)	Disc diffusion assay	6–9 mm inhibition zones for leaves, 5–9 mm for roots (depending on *S. aureus* strain)	Phenolic compounds	[85]
Anti-microbial	*Fragaria vesca* leaves	Hydroalcoholic extraction, separation	Disc diffusion assay	Good inhibition potential at 25 mg/mL, better effect for the ellagitannin-enriched fraction	Ellagitannins	[86]
Anti-allergenic	*Fragaria x ananassa* var. *Minomusume* fruits	Methanol fraction of fruits juice (obtained by squeezing)	Antigen-stimulated degranulation in RBL-2H3 cells	degranulation suppression (95–60% inhibition for linocinnamarin, cinnamic acid, chrysin, kaempferol, trans-tiliroside)	Best results - phenylpropanoid glycoside	[87]
Anti-diabetic	*Fragaria x ananassa* var. *Falandi* fruits	Compounds isolated from eth. extracts	α-glucosidase inhibitory activity	IC_50_ values better than the positive control (acarbose) for nine compounds (537.43 to 25.39 μM)	Individual compounds	[20]
Anti-obesity, anti-allergy, skin-lightening	*Fragaria ×ananassa* var. *Amaou,* entire plant (red fruit, green fruit, red calyx, green calyx, flower, leaf, stolon, stolon leaf, stem, crown and root)	Eth. or aq. room temperature extraction	Anti-lipase assay, adipocyte differentiation inhibition assay, melanogenesis inhibition assay, β-hexosaminidase inhibition assay, tyrosinase inhibition assay	Crown, stolon leaf and flowers extracts exhibited the highest effects	Total phenolic content	[35]
Antihyperuricemic	*Fragaria x ananassa* cv. *Tochiotome* leaves	Supercritical CO_2_ extraction with different entrainers	Uric acid production in AML12 hepatocytes	Reduction of uric acid at 100 mg/mL (96 mmol/2 h/mg protein), compared with the control (16,096 mmol/2 h/mg protein)	Kaempferol, quercetin	[10]
Cytotoxic, anti-proliferative	*Fragaria x ananassa* fruits	Meth. extraction	Ex vivo: cell viability assay; in vivo: developing tumor size determination	Cytotoxic on cancer cells, blocked the proliferation of tumor cells	Phenolic compounds	[89]
Antineoplastic	*Fragaria x ananassa* var. *Pajaro* fruits	Acidified hydro-eth. extraction	Transglutaminase assay and polyamine detection, immunoblot analysis	reduction of cell proliferation, lowering of the intracellular levels of polyamine, enhancement of tissue transglutaminase activity	Anthocyanins	[90]
Cytotoxic	*Fragaria vesca* L. leaves	Hydroalcoholic extract at room temperature, ellagitannins-enriched fraction	Effects on HepG2 cells—cell viability assessment, cell proliferation, cell cycle and cell death analysis, Western blot analysis, proteasome chymotrypsin-like activity	Inhibition of HepG2 cell viability IC_50_ = 690 mg/L (extract)/113 mg/L (fraction); fraction induced necrosis and apoptosis, influenced the cellular proteolytic mechanisms	Ellagitannins	[33]
Chemopreventive	Lyophilized *Fragaria x ananassa* fruits	Ultrasound-assisted extraction with acidified acetone	Histological studies, Western blot analysis, PGE_2_ measurement, and nitrate/nitrite colorimetric assay	Decreased tumor incidence, decreased levels of TNF-α, IL-1β, IL-6, COX-2 and iNOS, inhibition of the phosphorylation of PI3K, Akt, ERK, and NFκB	anthocyanins, ellagitannin/ellagic acid/ellagic acid derivatives flavonols	[95]
Cytotoxic	*Fragaria x ananassa* leaves	Hydroalcoholic extracts (meth., eth., isopropanol) from in vitro cell suspension	Cell proliferation, cell viability	Under 50% viable cells for colorectal adenocarcinoma and colon adenocarcinoma upon treatment with extracts containing 0.29 mM ethoxy-dihydrofuro-furan	Polyphenols	[91]

*where:* ADA—adenosine-deaminase; Akt—Protein Kinase B; aq.—water (aqueous); CAT—catalase; COX-2—cyclooxygenase-2 enzyme; ERK—extracellular signal-regulated kinase; eth—ethanol; GSH—glutathione; HepG2—human liver cancer cell line; IC_50—_half maximal inhibitory concentration; IL-1β—Interleukin 1 beta cytokine protein; IL-6—interleukin 6; iNOS—inducible nitric oxide synthase; meth.—methanol; MPO—myeloperoxidase; MTT—3-(4,5-dimethylthiazol-2-yl)-2,5-diphenyltetrazolium bromide; NFκB—nuclear factor kappa-light-chain-enhancer of activated B cells; NO—nitric oxide; PGE_2_—Prostaglandin E_2_; PI3K—phosphatidylinositol 3-kinase; RBL—rat basophilic leukemia cells; ROS—reactive oxygen species; SOD—superoxide dismutase; TNF-α—tumor necrosis factor alpha;.
